# Pathological Role of Interleukin-17 in Poly I:C-Induced Hepatitis

**DOI:** 10.1371/journal.pone.0073909

**Published:** 2013-09-19

**Authors:** Jianqin He, Guanjing Lang, Shiping Ding, Lanjuan Li

**Affiliations:** State Key Laboratory for Diagnosis and Treatment of Infectious Diseases, Department of Infectious Diseases, The First Affiliated Hospital, College of Medicine, Zhejiang University, Hangzhou, Zhejiang, China; French National Centre for Scientific Research, France

## Abstract

Immune-mediated responses were the main causes of liver damage during viral hepatitis, and recently viral RNA mimetic Poly I:C was used to induce a NK cell-dominated acute hepatitis. Interleukin-17A (IL-17A), the cytokine tightly associated with various autoimmune diseases, was known to play protective or pathological roles in LPS and ConA-induced hepatitis. However, its role in NK cell-mediated acute hepatitis remains unknown. Here we demonstrated that Poly I:C treatment triggered IL-17A production from hepatic γδT cells. Neutralizing IL-17A by monoclonal antibodies reduced Poly I:C-induced intrahepatic inflammatory responses and the liver injury through decreased accumulation, activation and cytolytic activity of NK cells in the liver. Furthermore, Poly I:C didn't trigger IL-17A secretion from γδT cells directly, and Kuppfer cells were demonstrated to be the accessory cell that can secrete IL-23. Finally, our findings demonstrated a pathological role of IL-17A and γδT cells in Poly I:C-induced acute hepatitis, which provides novel insights into viral infection-induced hepatitis and may serve as potential target in clinic immunotherapy against these disease.

## Introduction

Viral hepatitis is one of the most common health problems in the world, and HBV and HCV are the most prevailing viruses that specifically targeting the hepatocytes [1], [2]. However, HBV virus infection itself doesn't induce liver injury directly. The host immune responses triggered by the invading viruses are considered to be responsible for the liver injury [3], and previous studies have generally focused on virus specific T cells which are believed to mainly contribute to the liver damage under HBV infection [4], [5]. Natural killer (NK) cells are abundant in the liver and serve as a major innate immune component against various microbial infections [6], [7], especially virus infection. However, the role of NK cells in liver injury induced by HBV infection have been considered as an underinvestigated innate immune response [8]. Studies on viral hepatitis models in mice [9], [10] and human HBV patients [11], [12] have shown that NK cells may give rise to liver injury during viral infection. An acute hepatitis model induced by the viral RNA mimetic Polyinosinic-polycytidylic acid (Poly I:C) was demonstrated recently to emulate viral infection, which was suggested as the right model to study NK cell mediated liver injury [13], [14]. In the mouse liver, PolyI:C treatment causes the recruitment and activation of NK cells, a process dependent on Kupffer cell-mediated release of IL-12 and finally resulted in hepatocyte necrosis in the liver.

Proinflammatory cytokine IL-17A was originally identified from a subset of CD4 T cells that were named Th17 cells [15]. IL-17A induces neutrophils recruitment through the induction of cytokines which are important in granulopoiesis (G-CSF) and neutrophil chemotaxis (CXCL1 and CXCL8/IL-8) [16]. IL-17A and Th17 cells is essential for the development and pathogenesis of various autoimmune diseases such as multiple sclerosis, rheumatoid arthritis and inflammatory bowel disease [17], [18], and also protects against certain pathogens such as *Klebsiella pneumoniae* and *Candida* and *Mycobacterium tuberculosis* [19], [20]. In experimental hepatitis induced by *Listeria monocytogenes* infection, IL-17A was shown to increase the neutrophil accumulation in the liver and thus alleviated bacterial burden, paralleling with reduced liver damage [21], [22]. IL-17A-deficient mice injected with ConA developed a similar hepatitis as wild-type mice, which suggest that despite IL-17 being increased in T-cell-mediated hepatitis, it is dispensable in this model of liver injury [23]. Also, others reported that IL-17A either attenuated or aggravated the acute fulminate hepatitis induced by ConA injection [24], [25], [26]. Neutralizing IL-17A also decreased serum ALT and AST level in a toxic liver injury model induced by Halothane [27]. In addition, IL-17A is also shown to be involved in many types of human hepatitis, including alcohol induced liver injury and autoimmune hepatitis [28], [29]. Recently, IL-17A producing CD4 T cells are believed to play certain roles in HBV viral hepatitis, but the exact molecular and cellular pathways remain totally unknown [30]. Moreover, as NK cells are also involved in the pathogenesis of HBV hepatitis [11], [12], the role of IL-17A in NK cell mediated hepatitis and resultant liver injury remain largely unclear either.

In the present study, we employed Poly I:C to emulate viral infection and examined the effect IL-17A on NK cell-mediated liver injury. Poly I:C treatment triggered IL-17A production from liver γδT cells, which then exacerbated the inflammatory responses and liver damage through recruiting and activating of NK cells into the liver. Neutralizing IL-17A with monoclonal antibodies or depletion of γδT cells significantly attenuated acute hepatitis induced by Poly I:C injection. In addition, cytokines produced from Kuppfer cells may contribute to triggering IL-17A production from γδT cells. Our data have shown a pathological role of IL-17A and γδT cells in Poly I:C-induced acute hepatitis, which may serve as potential therapeutic target for viral hepatitis.

## Results

### Poly I:C induce IL-17 production from γδT cells

**Figure 1:**
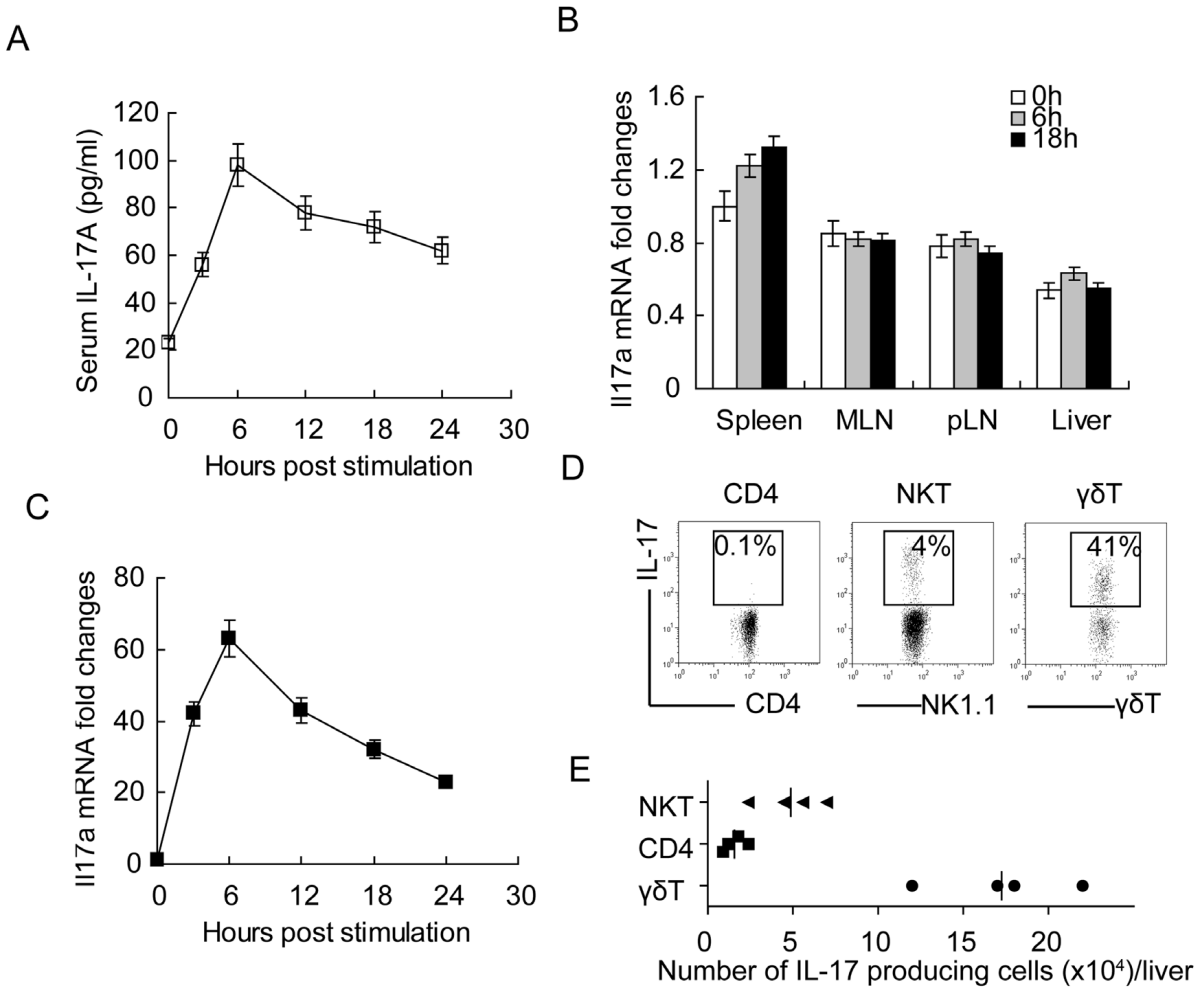
PolyIC induce IL17 production from liver γδT cells. (**A**) Serum IL-17 levels in male C57BL/6 mice at indicated time post PolyI:C stimulation. (**B**) Expression of Il17a mRNA in the spleen, MLN, pLN (peripheral lymph node) and liver after PolyI:C stimulation at 0 h, 6 h and 18 h. (**C**) Expression of Il17a mRNA in the mononuclear cell (MNC) isolated from the liver at indicated time after PolyI:C stimulation. (**D**) Flow cytometry intracellular staining of IL-17 in liver CD4 T cells, NKT cells and γδT cells. Plots were gated on CD4 T (CD3+CD4+) cells, NKT (NK1.1+CD3+) cells and γδT cells (γδTCR+) as indicated. The number shown was percentage of IL-17 positive cells in CD4, NKT or γδT cells. (**E**) Absolute number of IL-17 producing NKT cells, CD4 T cells and γδT cells in the liver. Data shown are representative of 3 independent experiments with similar results. Data shown are Means ± SD (A-C) or representative (D, E) from 3 independent experiments.

To investigate whether IL-17 is involved in NK cell-induced liver injury, we first tested the IL-17A expression in the serum after poly I:C challenge. In male C57 mice, serum IL-17 level was elevated after Poly I:C treatment, which reached the peak at 6 hours and then declined gradually (**Fig. 1A**). The same pattern was seen in female C57 mice and Balb/c mice (**Figure S1 and data not shown**), though slightly weak than male C57 mice. We then set to identify where these IL-17 came from. The whole RNA from the spleen, mesenteric lymph nodes (MLN) and liver was isolated and Il17a mRNA expression was examined. However, no significant increase of Il17a mRNA level was seen in these organ lyses after Poly I:C treatment, compared to their non-treated control (**Fig. 1B**). As the liver was composed mainly of hepatocytes which can't secret IL-17, we further isolated liver mononuclear cells (MNCs) by density gradient centrifugation. Though no increase of Il17a was seen in whole liver RNA, these hepatic leucocytes had elevated Il17a expression after Poly I:C challenge (**Fig. 1C**), implicating the liver as a major source of IL-17A in this model.

As previous studies suggested that CD4 T cells, NKT cells and γδT cells are the main sources of IL-17A and the liver contains all these cell types, we examined IL-17A expression in these cells respectively by intracellular cytokine staining. As shown in **Fig. 1D**, nearly half of the hepatic γδT cells produced IL-17A at 6 hours after Poly I:C treatment, while NKT cells produced less and CD4 T cells produced almost no IL-17A. The IL-17 producing cells in the liver was also quantitatively dominated by γδT cells (**Fig. 1E**). RT-PCR analysis showed elevated IL-17A mRNA expression in liver MNC with γδT cells after PolyI:C treatment, and γδT depletion greatly suppressed the Il17a upregulation (**Figure S2A**). These data indicated that Poly I:C can induce IL-17A secretion from liver γδT cells, suggesting a role of IL-17A in Poly I:C induced acute hepatitis. In addition, we also observed the upregulation of Il17f and Il22 mRNA in PolyI:C-treated liver MNC, which showed similar pattern with Il17a after γδT depletion. Neutralization of IL-17A didn't influence the expression of Il17f and Il22 mRNA (**Figure S2B**).

### IL-17A neutralization ameliorates Poly I:C-induced acute hepatitis

**Figure 2:**
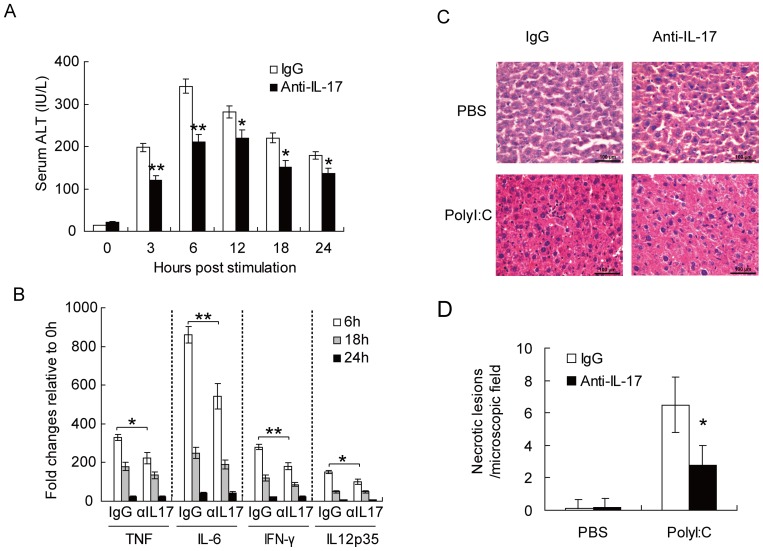
IL-17 neutralization attenuates PolyI:C induced acute hepatitis. (**A**) Serum ALT levels in mice neutralized of IL-17 or not at indicated times post PolyI:C stimulation. (**B**) Expression of cytokine mRNA level in liver MNC at 6 h, 18 h and 24 h post PolyI:C stimulation with or without IL-17 neutralization. αIL17: anti-IL-17. (**C**) Histological analysis of liver tissue from mice neutralized of IL-17 or not 8 h post PolyI:C stimulation. (**D**) Quantitative analysis of histological outcomes in (C): number of necrotic lesions in each microscopic field at a magnification of 200× (with 5 random microscopic fields per section). Data shown are Means ± SD (A-B,D) or representative (C) from 3 independent experiments.

To determine the protective or pathological role of IL-17A in Poly I:C-induced acute hepatitis, we neutralized IL-17A using anti-IL-17A monoclonal antibodies immediately after Poly I:C challenge. IL-17A neutralization after Poly I:C challenge resulted in reduced serum ALT level (**Fig. 2A**). We also examined the inflammatory cytokine gene expression in live leucocytes (**Fig. 2B**). Mice treated with isotype control antibody showed significant elevation of TNF, IL-6, IFN-γ, and IL-12 mRNA expression in liver leucocytes. However, reduced expression of these cytokines were observed in IL-17A neutralized mice challenged with Poly I:C. Histological analysis also showed significant hepatitis in Poly I:C challenged mice, as characterized by infiltrating of lymphocytes and hepatocyte apoptosis (**Fig. 2C**). In accordance, IL-17A neutralized mice displayed milder liver inflammation (**Fig. 2C, D**). These data suggested that neutralizing IL-17A can alleviate Poly I:C-induced acute liver injury.

### IL-17A contributes to NK cell accumulation and activation in Poly I:C-induced hepatitis

**Figure 3:**
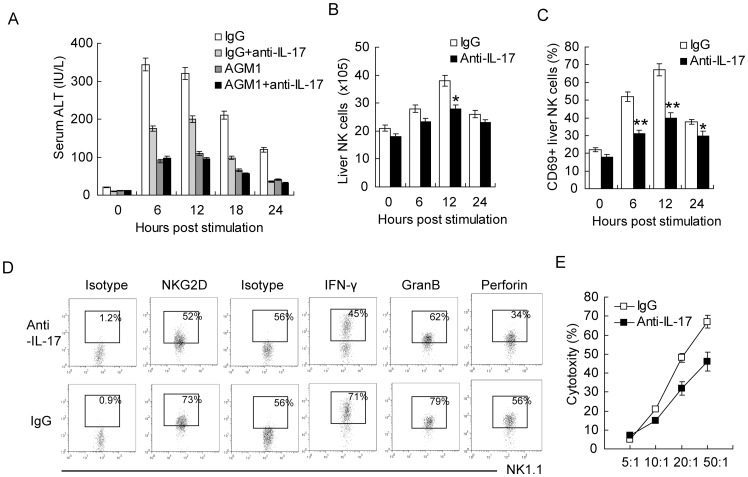
IL-17 promotes NK cell recruitment and activation in the liver. (**A**) Serum ALT levels in NK cell depleted mice at indicated times post PolyI:C stimulation. IL-17 was neutralized or not at the time of PolyI:C injection. (**B,C**) Absolute number of NK cells (B) and CD69 positive NK cells (C) at indicated time points in the liver after PolyI:C treatment, neutralized of IL-17 or not. (**D**) Flow cytometry staining of killing associated molecules (NKG2D, IFN-γ, Gramzyme B, perforin) expressed in NK cells 8 hours post PolyI:C treatment. Plots were gated on NKp1.1+CD3- cells. (**E**) Percentage of NK cell cytotoxity isolated from mice liver 8 hours after PolyI:C stimulation against YAC-1 cells. IL-17 was neutralized with monoclonal antibodies or not during the hepatitis. Data shown are Means ± SD (A-C,E) or representative (D) from 3 independent experiments. *P<0.05 **P<0.01.

Previous studies suggested that NK cells were the main immune cells damaging the hepatocytes, and contributing to direct liver injury in Poly I:C-induced acute hepatitis [13]. Thus, we depleted NK cells and then neutralized IL-17A after Poly I:C challenge. Without hepatic NK cells, the attenuation of ALT level by IL-17A neutralization was almost abrogated (**Fig. 3A**), suggesting that NK cells are indispensable in the exacerbation of Poly I:C-induced acute hepatitis by IL-17A. We next examined the NK cell accumulation and activation of these NK cells in the liver. As shown in **Fig. 3B**, Poly I:C-induced mild accumulation of NK cells in the liver, which was slightly inhibited by IL-17A neutralization, suggesting that IL-17A leads to NK cell recruitment in the liver after Poly I:C treatment. CD69 was used as a marker for NK cell activation, and significantly reduced CD69 positive NK cells were seen in IL-17A neutralized mice (**Fig. 3C**). We also analyzed the cell death inducing molecules of NK cells by flow cytometry. IL-17A neutralization reduced surface expression of NKG2D, the major killing ligand of liver NK cells (**Fig. 3D**). The secreted cell death inducing molecules IFN-γ, grazyme B and perforin were also reduced after IL-17A neutralization (**Fig. 3D**). We also detected the cytotoxity of these NK cells against YAC-1 target cells. NK cells neutralized of IL-17A showed reduced cytotoxity against YAC-1 cells (**Fig. 3E**). So, these data indicated that IL-17A not only contributes to the accumulation of NK cells in the liver, but also involved in the activation of liver infiltrating NK cells.

IL-17A was known to induce chemotaxis of neutrophils, and previous studies also suggested that IL-17A may exacerbate liver injury by recruitment of neutrophils [21]. However, the accumulation of neutrophils after Poly I:C injection was less significant than those with *Listeria monocytoegnes* infection, and the Ly6G+CD11b+ cells in the liver was not affected by IL-17A neutralization (**Figure S3**), indicating that the attenuation of Poly I:C-induced acute hepatitis by IL-17A neutralization was not caused by the reduction of neutrophil recruitment in the liver.

Previous studies have also shown a role of IL-12 for NK cell accumulation and poly I:C-induced liver injury [13]. IL-12 neutralization indeed ameliorated liver damage in the presence or absence of IL-17 (**Figure S4A**), which was in accordance with previous report. In the absence of IL-17, the whole number of NK cells in the liver was not significantly changed without IL-12, but NK cell activation was significantly suppressed (**Figure S4B,C**), suggesting for a critical role of IL-12 in PolyI:C-induced NK activation but not accumulation. We also analyzed the accumulation of γδT cells under IL-12 neutralization. As shown in **Figure S4D**, the amount of γδT cells remained much the same during PolyI:C-induced hepatitis, either with or without IL-12 neutralization.

### Depletion of γδT cells leads to reduced acute hepatitis

**Figure 4:**
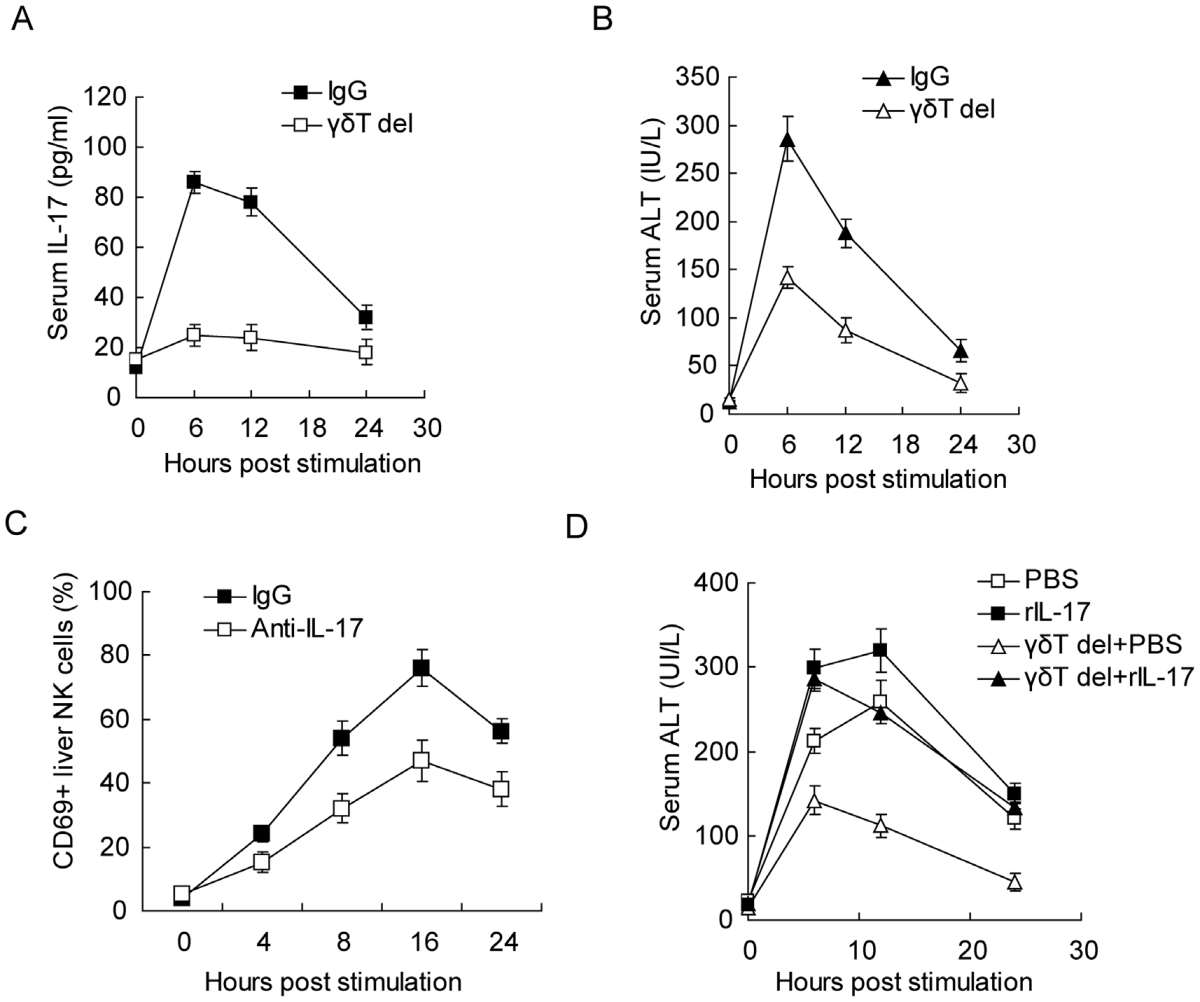
γδT cells contribute to PolyI:C induced hepatitis. (**A, B**) Serum IL-17 (A) and ALT (B) levels from PolyI:C treated mice depleted of γδT cells or not. (**C**) Analysis of NK cell activation in the liver depleted of γδT cells or not. (**D**) Serum ALT levels in PolyI:C treated mice with γδT cell depletion and then given exogenous rIL-17. Data shown are Means ± SD from 3 independent experiments.

Since a large percentage of γδT cells secreted IL-17A in Poly I:C-induced acute hepatitis (**Fig. 1D**), mice were depleted of these cells and then challenged with Poly I:C. As shown in **Fig. 4A**, depletion of γδT cells nearly abrogated IL-17A secretion in the serum, confirming that γδT cells were the main source of IL-17A. Accordingly, the liver injury identified by serum ALT level was also attenuated in γδT cell depleted mice (**Fig. 4B**). We also examined the accumulation and activation of NK cells after γδT cell depletion. Though the NK cell number in the liver was not significantly influenced, their activation as indicated by the percent of CD69+ NK cells did reduce after depletion of γδT cells (**Fig. 4C**). These data suggested that γδT cells can promote NK cell activation after Poly I:C challenge, and thereby exacerbating acute hepatitis. To elucidate the role of IL-17A in this process, we supplied recombinant mouse IL-17A intravenously into γδT cell-depleted mice after Poly I:C challenge, and found that rIL-17A to some extent abrogated the effect of γδT cell depletion, and significantly increased the serum ALT level (**Fig. 4D**). So, γδT cells may contribute to NK cell activation and subsequent liver injury by secreting IL-17A.

### Kuppfer cells contributes to the IL-17A secretion from γδT cells

**Figure 5:**
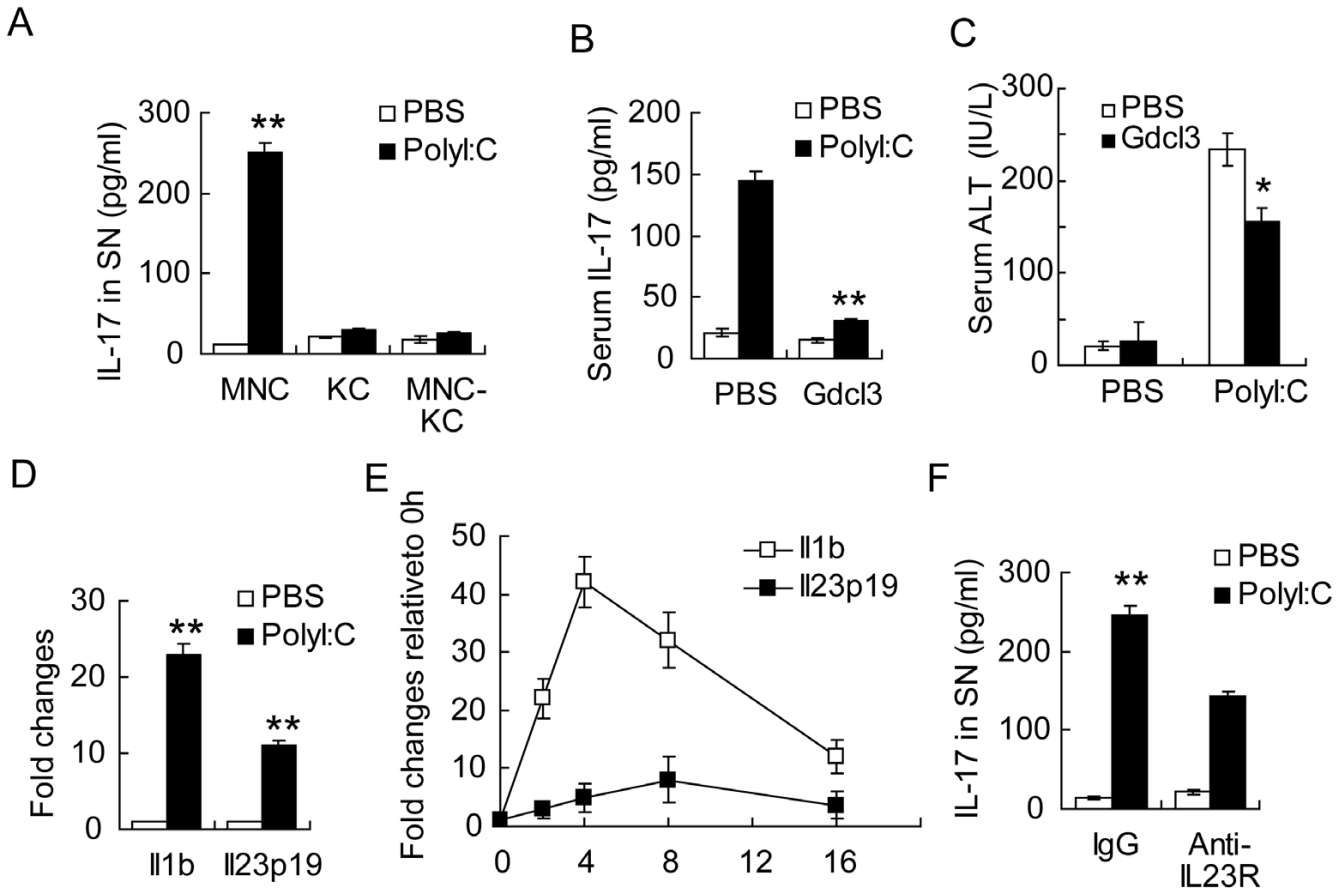
Involvement of Kupffer cells in the induction of IL-17 by PolyI:C. (**A**) ELISA assay of IL-17 in the supernatant (SN) of liver mononuclear cells (MNC), Kupffer cells (KC), and non-adherent cells (MNC-KC). SN: supernatant. (**B,C**) Serum IL-17 levels (**B**) and ALT (**C**) in PolyI:C treated mice depleted of Kupffer cells by pretreatment by Gdcl3. (**D**) RT-PCR of Il1b and Il23p19 mRNA expression in MNC isolated ex vivo from PolyI:C treated mice. (**E**) RT-PCR assay of Il1b and Il23p19 mRNA expression in MNC stimulated in vitro by PolyI:C. (**F**) ELISA of IL-17 in the supernatant of MNC stimulated with PolyI:C. IL-23R was blockaded by anti-IL-23R mAb (10 μg/ml) in some experiments before PolyI:C simulation. Data shown are Means ± SD from 3 independent experiments. *P<0.05 ** P<0.01.

We next determined how Poly I:C induced IL-17A production from hepatic γδT cells. As shown in **Fig. 5A**, Poly I:C induced substantail amout of IL-17A from whole hepatic mononuclear cells in vitro. Besides NK cells, Kuppfer cells abundantly present in the liver were also found to respond to Poly I:C stimulation, which may be responsible for the IL-17A production. To resolve the source of IL-17A, we cultured hepatic mononuclear cells in 37°C for 2 hours to allow Kuppfer cells to adhere to the plate. Then, the adherent cells and nonadherent cells were stimulated with Poly I:C, respectively. We observed no significant IL-17A production from either Kuppfer cells alone or the hepatic mononuclear cells depleted of Kuppfer cells (**Fig. 5A**), suggesting that Kuppfer cells are required for IL-17A production by γδT cells. *In vivo* depletion of Kuppfer cells by gadolinium chloride (Gdcl3) administration also confirmed that serum IL-17A and liver damage were greatly reduced after Poly I:C treatment in the absence of Kuppfer cells (**Fig. 5B, C**). These data suggested that Poly I:C couldn't trigger IL-17A production from γδT cells directly, and they need to corporate with Kuppfer cells.

Previous studies suggested that cytokines IL-23 and IL-1 could trigger IL-17A production from γδT cells. We first analyzed IL-1 and IL-23 mRNA expression in total hepatic mononuclear cells and Kuppfer cells. As shown in **Fig. 5D**, Poly I:C treatment indeed upregulated Il1b and Il23p19 transcription in liver mononuclear cells, which were also confirmed in vitro (**Fig. 5E**). Furthermore, neutralization of IL-23 inhibited IL-17A production from total hepatic mononuclear cells (**Fig. 5F**). These data indicated that IL-23 from Kuppfer cells elicited by Poly I:C stimulation may trigger γδT cells to produce IL-17A.

## Discussion

As an immune organ, the liver contains a lot of innate immune cells, including Kuppfer cells, NK cells and NKT cells, which act as the surveillant of the liver against bacterial and viral pathogens [6], [31]. However, overactivation of any of these cells can also lead to severe liver damage in an antigen nonspecific manner. NK cells are most abundant in the liver, constitute up to 50% of human intrahepatic lymphocytes [6]. Besides their anti-viral ability, many recent reports have taken notice of NK cell-mediated liver injury during viral hepatitis [9], [10], [11], [12], [14]. How to precisely constrain the activity of NK cells and thus restrict their cytotoxity against hepatocytes holds great significance for the control of acute fulminate hepatitis. Here, we demonstrated that IL-17A is pathogenic in NK cell-mediated liver injury after Poly I:C challenge. IL-17A production from γδT cells promoted NK cell accumulation in the liver, and facilitated their subsequent activation, which finally led to the exacerbated cytotoxity against hepatocytes.

The activity of IL-17A and Th17 cells in liver disease has been investigated extensively both in experimental liver injury models and human liver diseases [32], [33]. Patients with alcoholic liver disease have dramatically elevated serum IL-17A level, which mobilized neutrophil and led to acute alcohol liver disease through acting on hepatic stellate cells to trigger IL-8 and GRO-α production [28]. Several studies have suggested pro-tumor effect of Th17 cells or IL-17A in hepatic tumor formation and progression, shortening patient survival by promoting tumor angiogenesis [34], [35], [36]. Th17 cells are also highly enriched in both peripheral blood and liver during chronic HBV viral infection, and exhibit a potential to exacerbate liver damage [30]. In the current study, we demonstrated the pathological role of IL-17A on NK cell-mediated liver injury. IL-17A promotes the mobilization and cytotoxity of hepatic NK cells. Recently, Kawakami et al has reported that IL-17A inhibits skin NK cell's activity in a mouse model of atopic dermatitis, which was apparently at odds with our reports [37]. However, in that eczema vaccinatum model, skin NK cells were activated in a IL-4 dependent way, while in Poly I:C-induced hepatitis, hepatic NK cells were activated by Poly I:C directly and the inflammatory cytokines IL-12 and type I IFN [38], [39], [40], [41]. Different activation routes of NK cells and different immune microenvironment of the response may lead to different regulatory mechanisms of these NK cells. In addition, IL-17A may also promote the virus survival by suppressing apoptosis of the infected cell through upregulating anti-apoptotic molecules [42], [43]. However, we also detected milder expression of IL-17RA in NK cells (**Figure S5**), and therefore can't exclude the possibility that IL-17 may also exacerbate PolyI:C-induced liver damage through acting directly on NK cells.

Activated CD4+ T cells were initially deemed as the major producers of IL-17. However, several other studies suggest γδT cells can be the main source of IL-17 under certain circumstances, especially in the liver [21], [22], [44]. Differing from the conventional induced Th17 cells, IL-17–producing γδT cells seem to differentiate in the thymus and their activation is TCR independent [45]. In the periphery, IL-17 production from γδT cells can be triggered by IL-23 and other inflammatory factors such as IL-1β [46]. In other studies, the subsets of IL-17A-producing γδT cells have been investigated extensively, which are restricted to the Vγ4 and Vγ6Vδ1 subsets [21]. Identically, in Poly I:C-induced liver injury, IL-17A production in the liver still come from a subset of γδT cells. The detailed mechanism on how IL-17A is induced in these γδT cells is yet to be fully elucidated. Nevertheless, it is clear from our study that Kuppfer cells are indispensable in this process, as depletion of Kuppfer cells both *in vitro* and *in vivo* leads to the loss of IL-17A production from intrahepatic lymphocytes by Poly I:C stimulation. Poly I:C induces IL-1 and IL-23 mRNA transcription in liver Kuppfer cells. Neutralization of IL-23 also attenuates IL-17A production, indicating a role of IL-23 in facilitating the effect of Kuppfer cells. Recently, Wang et al have reported the detrimental role of IL-17A in the side effect of a commonly used over-the-counter drug, acetaminophen [47]. In this neutrophil dominated hepatitis model, the HMGB1-TLR4-IL-23 pathway in macrophages regulates the generation of IL-17-producing γδT cells, which was in accordance with our PolyI:C-induced IL-17 production. There are also publications about IL-17 secreting NK cells in a mouse infection model, where IL-17 secretion is IL-6 dependent [48]. However, we had no evidence that IL-6 is involved in the IL-17 production in our model. We blocked IL-6 in the cultured liver MNCs stimulated with PolyI:C in vitro, but didn't find significant decrease of IL-17 production (**Figure S6**).

IL-17A can stimulate the cytokine/chemokine expression (G-CSF, CXCL1 and CXCL8/IL-8), which are important in the mobilization and activation of PMN cells [16]. Many reports have suggested that the exacerbated liver injury by IL-17A in various hepatitis models can be attributing to its ability to recruit neutrophils [21], [49], [50]. However, we have not seen abundant PMN infiltration into the liver after Poly I:C treatment, indicating PMN may not be critically involved in this NK-mediated hepatitis. In addition, there is no difference of PMN numbers in the liver neutralized of IL-17A or not. IL-17F can also signal through IL-17RA and is known to compensate for IL-17A function [51], and Il17f mRNA in PolyI:C treated liver MNC showed similar pattern with IL-17A. These results suggested that PolyI:C-induced IL-17F may have synergistic effect with IL-17A. Moreover, IL-22 was induced together by PolyI:C, which was previously shown to have protective effect on hepatocyte apoptosis [23], [52]. This opposing effect of IL-22 and IL-17A/F may underlie the precise homeostasis and dynamic balance of the immune system.

In conclusion, we showed here that IL-17A is involved in the pathogenesis of Poly I:C-induced liver injury by promoting the NK cells accumulation and activation of NK cells in the liver. Poly I:C treatment triggered IL-17A production from liver γδT cells. Neutralizing IL-17A with monoclonal antibodies or depletion of γδT cells significantly attenuated acute hepatitis induced by Poly I:C injection. Thus,we revealed the pathological role of IL-17A and γδT cells in Poly I:C-induced acute hepatitis, which provides novel insights into viral infection-induced hepatitis and may serve as potential target in clinic immunotherapy against these diseases.

## Materials and Methods

### Mice and Reagents

C57BL/6 mice and Balb/c were purchased from Zhejiang University Laboratory Animal Center, maintained and used in accordance with the institutional guidelines for animal care. Anti-CD4 (L3T4), anti-CD8 (Ly2), anti-IFN-γ (XMG1.2), anti–IL-17A (TC11-18H10) was purchased from BD pharmingen (San Diego, CA). anti-IL-23Rα antibodies (MAB1686), anti-mouse IL-17A mAb (MAB421), anti-IL-12 (C17.8), anti-IL-6 (MP5-20F3) were from R&D Systems. Recombinant mouse IL-17A was from BioLegend.

### Poly I:C induced hepatitis model

Mice were intraperitoneally injected with 30 μg/g body weight Poly I:C. At indicated time, mice were sacrificed. Serum were collected for alanine aminotransferase (ALT) detection and ELISA assay, and liver histology were assessed to represent disease severity. Single cell suspensions of spleen, or liver tissues were also prepared in some circumstance for flow cytometry analysis.

### Splenic and Hepatic mononuclear cell Isolation

Single cell suspensions of the spleen and lymph nodes were obtained by gently squeezing tissues through a wire mesh screen with cold RPMI medium. Red blood cells were lysed with Tris-NH4Cl solution. Hepatic mononuclear cells (MNC) were isolated following a previously described method with slight modification [24]. Mice were anesthetized and the liver was perfused in situ with Hank's balanced salt solution (HBSS) pre-warmed at 37°C for 5 min. Single cell suspensions were generated by filtering the liver through a 100 μm cell strainer (BD Falcon, Bedford, MA). After washing, the pellet was re-suspended in 15 mL of 35% Percoll (Sigma) containing 50 U/mL of heparin and centrifuged at 500×g for 15 min. The resulting pellet was collected and resuspended in Tris-NH4cl for 5 min, then washed in HBSS solution and used as liver MNCs. Total viable hepatic leukocytes were counted by trypan blue exclusion.

### Cytotoxicity assay of NK cells

NK cells's cytotoxicity was determined by 51Cr release assay as previously described [13]. YAC-1 cells were used as target cells. The liver MNCs were used as effector cells. Target cells were incubated with effector cells at various E:T ratios. Specific lysis (%) was determined as follows: 100× (experimental release-spontaneous release)/(maximum release-spontaneous release).

### RNA isolation and Real-time PCR

RNA was isolated using RNAfast200 (Fastagen, China) according to the manufacturer's instructions. IL-17A, TNF, IL-6, and IL-12 were quantified by real-time RT-PCR. β-actin was used as endogenous control. A 2^−ΔΔCt^ method was used to calculate fold change. The primer sequences used in this study are shown in **Table S1**. Amplified products were monitored directly by measuring the increase of the dye intensity of the SYBR Green I (Molecular Probes, Eugene, OR) that binds to the double-strand DNA amplified by PCR.

### Intracellular cytokine staining and flow cytometry

Surface staining was performed as described previously. For intracellular cytokine staining, cells were stimulated with 25 ng/ml PMA and 500 ng/ml ionomycin (Sigma-Aldrich, St. Louis, MO) for 6 h at 37°C. Brefeldin A (10 mg/ml; eBioscience) was included for the last 4 h of incubation. Cells were stained with the Cytofix/Cytoperm kit according to the manufacturer's instructions (eBioscience).

### Cell depletion and IL-17A neutralization

Mice were administered with an i.v. injection of 20 μl anti-asialo GM1 (Wako Pure Chemicals, Osaka, Japan) diluted in 200 μl pyrogen-free PBS to deplete NK cells 2 days before Poly I:C challenge. To deplete γδT cells, we injected mice i.v. with 200 μg anti-γδTCR mAb (UC7-13D5, Biolegend) diluted in 200 μl pyrogen-free PBS. Kupffer cells were depleted with i.v. injection of 10 mg/kg Gdcl3 1 days before experiments. Depletion was confirmed by flow cytometry. For neutralizing effect of IL-17A *in vivo*, 100 μg/mouse anti-mouse IL-17A mAb (MAB421; R&D Systems) or rat IgG2a isotype control was injected i.v. 1 h before Poly I:C treatment.

### Measurement of IL-17 level

The IL-17 level in the serum and supernatant was measured by ELISA according to the manufacturer's instructions (R&D Systems, Minneapolis, MN).

### Statistical Analysis

Data are presented as mean ± SD. Student's t-test was used to compare two groups. Comparisons among multiple groups were performed using one-way analysis of variance (ANOVA) with a post-hoc test of significance between individual groups. Differences were considered significant when p<0.05.

## Supporting Information

Figure S1 **1 Serum IL-17 levels in female C57 mice post Poly I:C challenge.** Female C57BL/6 mice were stimulated with Poly I:C i.p., and serum was collected at indicated times and IL-17 levels were measured by ELISA. Data shown were Means ± SD from three independent experiments.
(DOC)

Figure S2 **Expression of Il17, IL17f and Il22 in liver after depletion of γδT cells or neutralized of IL-17.** (**A**) Expression of Il17a, Il17f and Il22 mRNA in liver mononuclear cell (MNC) isolated from wild type mice or mice depleted of γδT cells 6 hours after PolyI:C stimulation. (**B**) RT-PCR analysis of Il17f and Il22 mRNA in liver MNC neutralized of IL-17A or not. Data shown were Means ± SD from 3 independent experiments. *P<0.01.
(DOC)

Figure S3 **Neutrophils accumulation in the liver and spleen in Poly I:C stimulated mice after IL-17 neutralization.** C57BL/6 mice were stimulated with Poly I:C i.p., and infiltrating neutrophils were detected by flow cytometry in the spleen and liver 8 hour post treatment. Data shown were representative from two independent experiments.
(DOC)

Figure S4 **Influence of IL-12 on PolyI:C induced hepatitis.** Serum ALT levels (**A**), absolute number of NK cells in the liver (**B**), CD69+ NK cells (**C**) and liver γδT cell number (**D**) were determined 6 hours post PolyI:C challenge in the presence or absence of IL-17. Anti-IL12 (100 μg/mouse) or control IgG was blocked as indicated before PolyI:C injection. Data shown were Means ± SD from three independent experiments. *P<0.05 ** P<0.01.
(DOC)

Figure S5 **IL-17RA expression on NK cells.** Liver MNCs were isolated from C57 mice and stained with anti-IL-17RA or isotype control mAb for FACS analysis. Data shown are representative of 2 independent experiments with similar results. Data shown were representative from two independent experiments.
(DOC)

Figure S6 **Influence of IL-6 on IL-17 production from liver MNCs.** Liver MNCs were isolated and stimulated with PolyI:C for 24 hours. Supernatant was collected and assayed with ELISA for IL-17 concentration. Anti-IL-6 or isotype IgG control was added as indicated. Data shown were Means ± SD from three independent experiments.
(DOC)

Table S1 **Real time PCR Primers sequences.**
(DOC)
