# Supplementary material for: Pathological Role of Interleukin-17 in Poly I:C-Induced Hepatitis
Source: PLoS One. 2013 Sep 19;8(9):e73909. doi: 10.1371/journal.pone.0073909 (PMC3777971; doi:10.1371/journal.pone.0073909)
Supplement: Table S1 — Real time PCR Primers sequences. (DOC) [file pone.0073909.s007.doc]

| Gene symbol | Primer sequence (5’🡪3’) | |
| --- | --- | --- |
| Tnf | Forward | GACGTGGAACTGGCAGAAGAG |
|  | Reverse | TTGGTGGTTTGTGAGTGTGAG |
| Il17a | Forward | TTTAACTCCCTTGGCGCAAAA |
|  | Reverse | CTTTCCCTCCGCATTGACAC |
| Il17f | Forward | TGCTACTGTTGATGTTGGGAC |
|  | Reverse | AATGCCCTGGTTTTGGTTGAA |
| Il22 | Forward | ATGAGTTTTTCCCTTATGGGGAC |
|  | Reverse | GCTGGAAGTTGGACACCTCAA |
| Il6 | Forward | CCAAGAGGTGAGTGCTTCCC |
|  | Reverse | CTGTTGTTCAGACTCTCTCCCT |
| Il12a | Forward | CTGTGCCTTGGTAGCATCTATG |
|  | Reverse | GCAGAGTCTCGCCATTATGATTC |
| Beta-actin | Forward | GCTGCGTTTTACACCCTTTC |
|  | Reverse | GCTGTCGCCTTCACCGTTC |
