# Supplementary material for: Pathological Role of Interleukin-17 in Poly I:C-Induced Hepatitis
Source: PLoS One. 2013 Sep 19;8(9):e73909. doi: 10.1371/journal.pone.0073909 (PMC3777971; doi:10.1371/journal.pone.0073909)
Supplement: Supplementary file 2 [file pone.0073909.s001.doc]

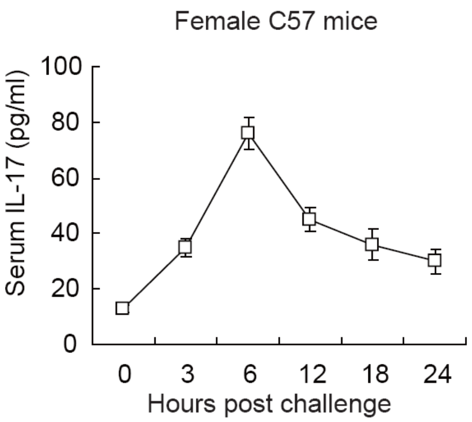
Female C57 mice
Serum IL-17 (pg/ml)
100
80
60
40
20
0
0
3
6
12
18
24
Hours post challenge
