# Supplementary material for: Pathological Role of Interleukin-17 in Poly I:C-Induced Hepatitis
Source: PLoS One. 2013 Sep 19;8(9):e73909. doi: 10.1371/journal.pone.0073909 (PMC3777971; doi:10.1371/journal.pone.0073909)
Supplement: Supplementary file 3 [file pone.0073909.s002.doc]

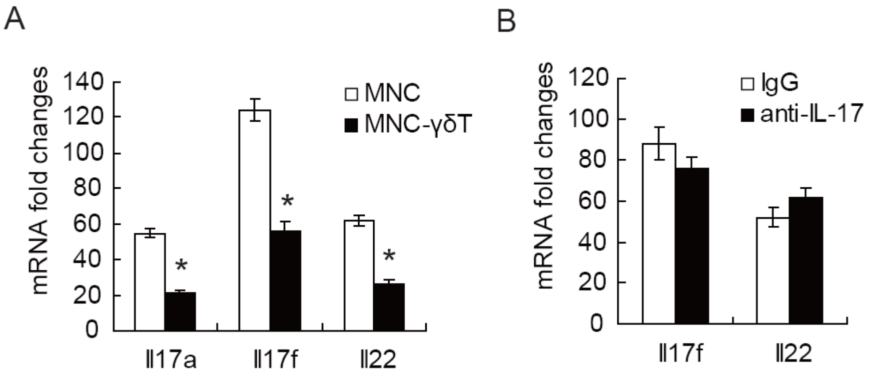

A
mRNA fold changes
140
120
100
80
60
40
20
0
MNC
MNC-γδT
*
*
*
Il17a
Il17f
Il22
B
mRNA fold changes
120
100
80
60
40
20
0
IgG
anti-IL-17
Il17f
Il22
