# Supplementary material for: Pathological Role of Interleukin-17 in Poly I:C-Induced Hepatitis
Source: PLoS One. 2013 Sep 19;8(9):e73909. doi: 10.1371/journal.pone.0073909 (PMC3777971; doi:10.1371/journal.pone.0073909)
Supplement: Supplementary file 4 [file pone.0073909.s003.doc]

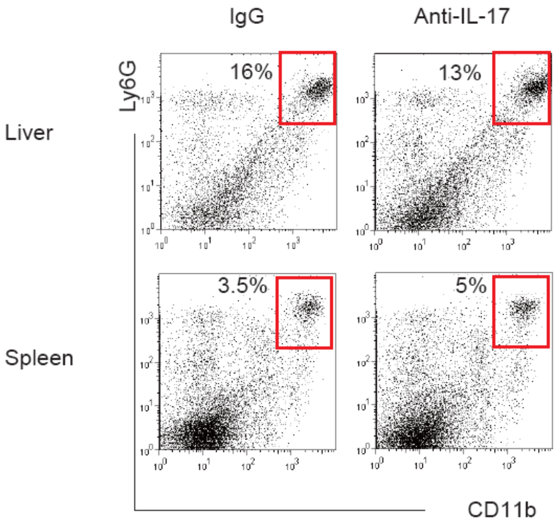
IgG
Anti-IL-17
Liver
Spleen
Ly6G
CD11b
16%
13%
3.5%
5%
