# Supplementary material for: Pathological Role of Interleukin-17 in Poly I:C-Induced Hepatitis
Source: PLoS One. 2013 Sep 19;8(9):e73909. doi: 10.1371/journal.pone.0073909 (PMC3777971; doi:10.1371/journal.pone.0073909)
Supplement: Supplementary file 5 [file pone.0073909.s004.doc]

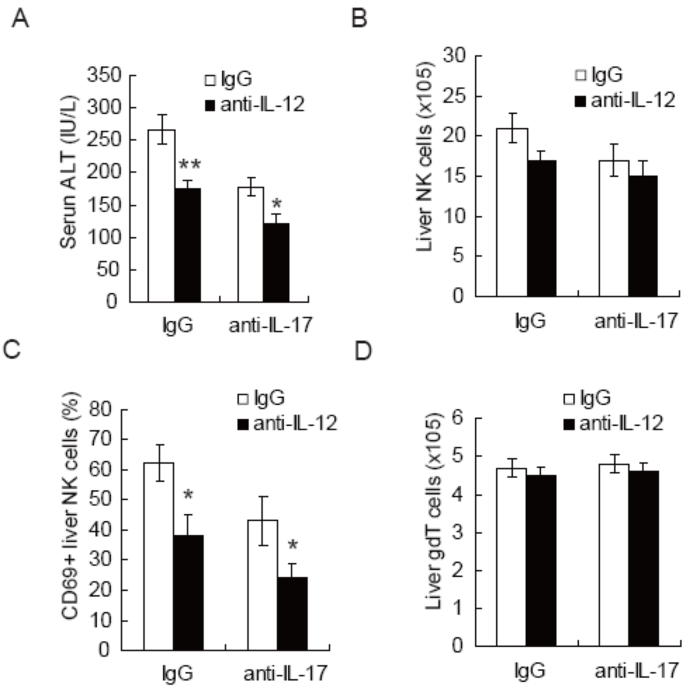
A
Serun ALT (IU/L)
IgG
anti-IL-12
IgG
anti-IL-17
B
Liver NK cells (x105)
IgG
anti-IL-12
IgG
anti-IL-17
C
CD69+ liver NK cells (%)
IgG
anti-IL-12
IgG
anti-IL-17
D
Liver gdT cells (x105)
IgG
anti-IL-12
IgG
anti-IL-17
