# Supplementary material for: Pathological Role of Interleukin-17 in Poly I:C-Induced Hepatitis
Source: PLoS One. 2013 Sep 19;8(9):e73909. doi: 10.1371/journal.pone.0073909 (PMC3777971; doi:10.1371/journal.pone.0073909)
Supplement: Supplementary file 6 [file pone.0073909.s005.doc]

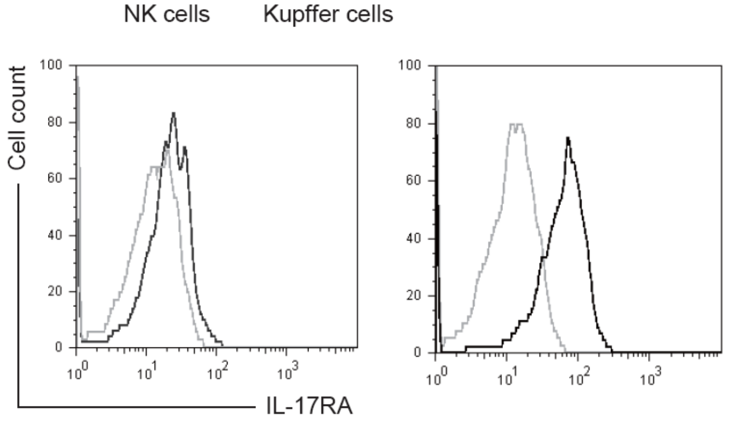
NK cells
Kupffer cells
Cell count
IL-17RA
100
80
60
40
20
0
10^0
10^1
10^2
10^3
