# Supplementary material for: Pathological Role of Interleukin-17 in Poly I:C-Induced Hepatitis
Source: PLoS One. 2013 Sep 19;8(9):e73909. doi: 10.1371/journal.pone.0073909 (PMC3777971; doi:10.1371/journal.pone.0073909)
Supplement: Supplementary file 7 [file pone.0073909.s006.doc]

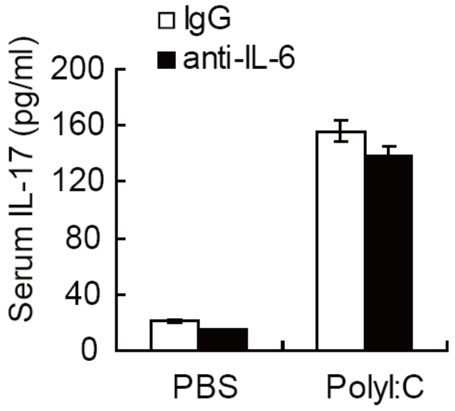
IgG
anti-IL-6
Serum IL-17 (pg/ml)
200
160
120
80
40
0
PBS
PolyI:C
